# Tongxinluo enhances the effect of atorvastatin on the treatment of atherosclerosis with chronic obstructive pulmonary disease by maintaining the pulmonary microvascular barrier

**DOI:** 10.1002/fsn3.3070

**Published:** 2022-10-13

**Authors:** Xiangnan Kuang, Yafen Wang, Shiqiao Liu, Liping Chang, Yujie Yin, Zhen Li, Yi Liu, Wenyan Li, Yunlong Hou, Hongtao Wang, Junqing Liang, Zhenhua Jia

**Affiliations:** ^1^ Hebei University of Chinese Medicine Shijiazhuang China; ^2^ Hebei Key Laboratory of Integrated Chinese and Western Medicine for Lung Disease Research Shijiazhuang China; ^3^ Key Laboratory of State Administration of Traditional Chinese Medicine (Cardio‐Cerebral Vessel Collateral Disease) Shijiazhuang China; ^4^ Hebei Yiling Pharmaceutical Research Institute Shijiazhuang China; ^5^ Graduate School Hebei Medical University Shijiazhuang China; ^6^ National Key Laboratory of Collateral Disease Research and Innovative Chinese Medicine Shijiazhuang China; ^7^ Department of Cardiology Affiliated Yiling Hospital of Hebei University of Chinese Medicine Shijiazhuang China

**Keywords:** atherosclerosis, atorvastatin, chronic obstructive pulmonary disease, pulmonary microvascular barrier, tongxinluo

## Abstract

Atherosclerosis (AS) is a common comorbidity of chronic obstructive pulmonary disease (COPD), and systemic inflammation is an important mechanism of COPD with AS. Tongxinluo (TXL) improves the function of vascular endothelial cells. We aimed to prove that impairment of pulmonary microvascular barrier function is involved in COPD‐mediated aggravation of AS and investigate whether TXL enhances the effect of Ato (atorvastatin) on COPD with AS by protecting pulmonary microvascular endothelial barrier function. In vivo, a COPD with atherosclerotic apolipoprotein E knockout (AS ApoE^−/−^) mouse model was established by cigarette smoke combined with a high‐fat diet. The animals were administered TXL, Ato, and TXL + Ato once a day for 20 weeks. Lung function, lung microvascular permeability, lung inflammation, systemic inflammation, serum lipid levels, atheromatous plaque formation, and endothelial damage biomarkers were measured. In vitro, human pulmonary microvascular endothelial cells (HPMECs) were pretreated with TXL and incubated with cigarette smoke extract to establish the model. The permeability of the endothelial monolayer, inflammatory cytokines, endothelial damage biomarkers, and tight junction (Tj) proteins were determined. Cigarette smoking significantly exacerbated the high‐fat diet‐induced pulmonary function decline, pulmonary microvascular endothelial barrier dysfunction, inflammation, and atherosclerotic plaques. These changes were reversed by TXL–Ato; the combination was more effective than Ato alone. Furthermore, TXL protected the HPMEC barrier and inhibited inflammation in HPMECs. COPD aggravates AS, possibly through the destruction of pulmonary microvascular barrier function; thus, lung inflammation triggers systemic inflammation. In treating COPD with AS, TXL enhances the antiatherosclerotic effect of Ato, protecting the pulmonary microvascular barrier.

## Graphical abstract

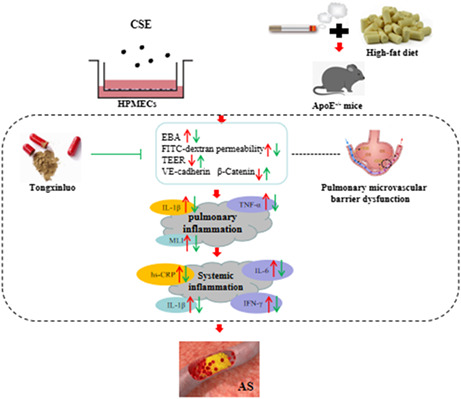

## 1 INTRODUCTION

Chronic obstructive pulmonary disease (COPD) is characterized by an incomplete reversible airflow restriction, which gradually worsens and is associated with abnormal inflammatory responses to harmful gases or particles in the lungs, such as smoke from tobacco and biomass combustion (GOLD, 2019; Labaki & Rosenberg, 2020). Over 300 million people suffer from COPD. Currently, this condition is the fourth leading cause of death worldwide and is predicted to be ranked third by 2030 (WHO, 2010). In addition to pulmonary symptoms, COPD is accompanied by extrapulmonary comorbidities, such as cardiovascular disease (CVD), metabolic syndrome, and diabetes (Choudhury et al., 2014; Wenjia et al., 2015). CVD is a common comorbidity and a major cause of death in patients with COPD (Curkendall et al., 2006; Huiart et al., 2005), indicating their epidemiological link (Eriksson et al., 2013; Mullerova et al., 2013). Accumulating evidence has shown that systemic inflammation is associated with COPD, which may underlie the increased risk of COPD combined with atherosclerosis (AS; Fabbri et al., 2008; Fuschillo et al., 2012; Roversi et al., 2014; Sin & Man, 2003).

Previous studies have shown that increased pulmonary microvascular permeability may be a key factor for airway obstruction in patients with COPD (Kyomoto et al., 2019). The increased permeability of pulmonary microvascular endothelial cells (PMVECs) causes neutrophil accumulation and subsequent activation in the lungs. These cytokines stimulate additional inflammatory cells and facilitate the release of inflammatory mediators, which in turn trigger inflammatory cascades and amplify injury signals to cause systemic inflammation. Importantly, systemic inflammation plays a crucial role in the pathogenesis of AS (Baumer et al., 2018). Therefore, maintaining the integrity of the pulmonary microvascular endothelial barrier and preventing pulmonary inflammation from leading to systemic inflammation are key factors in the treatment of COPD combined with AS. However, the role of pulmonary microvascular barrier function in the progression of COPD with AS has not yet been elucidated due to the lack of relevant studies. The treatment regimen for patients with COPD combined with AS involves the treatment of symptoms for a single disease, including inhalation of bronchodilators and anti‐inflammatory therapy for COPD (Halpin David et al., 2021), lipid‐lowering treatment (for example, statins), antihypertensive agents, or β‐blockers for AS (Baigent et al., 2005). The symptoms of both diseases are treated as independent modalities, and the interactions between COPD and AS are not considered. Therefore, new treatment regimens for COPD combined with AS are required.

Tongxinluo (TXL) is a Chinese herbal compound that has been widely used in the treatment of CVDs and cerebrovascular diseases in China (Chen et al., 2009; Zhang et al., 2009). TXL is composed of*Radix ginseng*, *Buthus martensii*, *Hirudo*, *Eupolyphaga seu steleophaga*, *Scolopendra subspinipes*, *Periostracum cicadae*, *Radix paeoniae rubra*, *Semen ziziphi spinosae*, *Lignum dalbergia odoriferae*, *Lignum santali albi*, and *Borneolum syntheticum* (Chang et al., 2017). To date, approximately 6 million patients with CVDs and cerebrovascular diseases have been treated with TXL due to its beneficial effects against vascular diseases. Our previous studies showed that TXL not only relieves the symptoms of chest tightness and chest pain and significantly inhibits oxidized low‐density lipoprotein (ox‐LDL)‐induced apoptosis of macrophages by enhancing autophagy (Chen, Li, Zhang, et al., 2018), but also alleviates the lung symptoms of dyspnea, significantly increasing the 6‐min walk distance (6MWD) and the level of the lung marker Clara cell secretory protein‐16 (CC‐16; Liu et al., 2019) in patients with COPD with CVD. Previous studies have shown that TXL protects human cardiac microvascular endothelial cells from hypoxia/reoxygenation injury and alleviates cerebral microcirculatory disturbances against ischemic injury. Furthermore, TXL improved the renal structure and function of individuals with diabetic nephropathy (Cui et al., 2014; Liu et al., 2018; Wang et al., 2014). TXL‐mediated improvement in microvascular blood flow perfusion is a common mechanism in the treatment of major diseases of the heart and brain and diabetic nephropathy, and its target is mainly microvascular endothelial cells. However, the role of TXL in PMECs has not been reported. The antiatherosclerotic effects and concomitant lipid‐lowering effects of atorvastatin (Ato) have been demonstrated in previous studies (Meredith et al., 2005). The present study aimed to determine whether the key mechanism of COPD exacerbation of AS is pulmonary microvascular barrier dysfunction, whether lung inflammation triggers systemic inflammation, and whether TXL can enhance the antiatherosclerotic effect of Ato in COPD combined with AS through pulmonary microvascular barrier protection.

## 2 MATERIALS AND METHODS

### 2.1 Ethics statement

Adult male wild‐type (WT) mice with a C57BL/6N background and apolipoprotein E knockout (ApoE^−/−^) mice (Whitman, 2004; Zhang et al., 1992; 20–25 g; 8–10 weeks old) were purchased from Beijing Vital River Laboratory Animal Technology Co., Ltd. All animals were kept at the Key Laboratory of State Administration of Traditional Chinese Medicine (Cardio‐Cerebral Vessel Collateral Disease). All procedures with animals were conducted under the guidelines on the Care and Use of Laboratory Animals for biomedical research published by the National Institutes of Health (No. 85‐23, revised 1996) and according to the ethical guidelines of the Ethics Committee of Hebei Yiling Pharmaceutical Research Institute (No. N2020051).

### 2.2 Animals and treatments

ApoE^−/−^ mice with a C57BL/6N background were used as an animal model of human AS. Knockout of the ApoE allele results in hypercholesterolemia, increasing the susceptibility of the mice to AS when they are fed a high‐fat diet. To eliminate the effect of a high‐fat diet on the mouse lungs, we used C57BL/6N mice without spontaneous AS in this study. All mice were housed in an animal center under a 12‐h light–dark cycle for 7 days to acclimatize to the environment prior to experimentation. The animals had free access to a standard rodent diet and water at all times. According to preliminary experiments, the dose of TXL was determined to be 1.5 g/kg/day (approximately 2 times the clinically equivalent dose; Chen, Li, Zhang, et al., 2018), and that of Ato was 10 mg/kg/day (Han et al., 2016). As previously described (Arunachalam et al., 2010; Florence et al., 2018), the smoke was pumped into a plastic box, measuring 42 cm (length) × 28 cm (width) × 27 cm (height), containing animals that passively inhaled the cigarette smoke. AS was induced in ApoE^−/−^ mice fed a high‐fat diet (SCXK2019‐0003; Beijing Keao Xieli Feed Co., Ltd.). The detailed grouping and treatment of the animals are shown in Table 1.

**TABLE 1:** Grouping and treatment schedule

	Group (*n* = 10)	Species	Dose, route, treatment, and duration	Frequency
1	Control	C57BL/6N	0.5% carboxymethyl cellulose (CMC) by gavage, normal chow diet, ambient air for 20 weeks	—
2	AS	ApoE^−/−^	0.5% CMC by gavage, high‐fat diet, ambient air for 20 weeks	—
3	COPD + AS		0.5% CMC by gavage, high‐fat diet, cigarette smoke for 20 weeks	Cigarette smoke: twice a day for 1 h/20 cigarettes/time, 5 days/week
4	COPD + AS + TXL (TXL)		Tongxinluo (TXL; 1.5 g/kg/day) by gavage, high‐fat diet, cigarette smoke for 20 weeks	
5	COPD + AS + Ato (Ato)		Atorvastatin (Ato; 10 mg/kg/day) by gavage, high‐fat diet, cigarette smoke for 20 weeks	
6	COPD + AS + TXL + Ato (TXL + Ato)		TXL (1.5 g/kg/day) + Ato (10 mg/kg/day) by gavage, high‐fat diet, cigarette smoke for 20 weeks	

### 2.3 Preparation of TXL

Tongxinluo ultrafine powder (Shijiazhuang Yiling Pharmaceutical Co.) was dissolved in 0.5% sodium carboxymethyl cellulose (CMC) solution and stirred with a magnetic stirrer for 1 h. The suspension was intragastrically administered to the mice daily at a dose of 1.5 g/kg (volume: 0.1 ml/10 g) per day by oral gavage.

In addition, TXL ultrafine powder was solubilized in TXL in serum‐free Dulbecco's modified Eagle's medium (DMEM) and sonicated. The pellet was collected by centrifugation at 10,000× g for 10 min. Subsequently, the precipitate was dried at 60°C to obtain the drug mass that is not dissolved in DMEM culture medium, and the supernatant was filtered (0.22‐μm pore size, Costar) to calculate an accurate weight of the dissolved ingredients (Chang et al., 2017). According to previous reports (Cui et al., 2014; Yin et al., 2019) and MTS [3‐(4,5‐dimethylthiazol‐2‐yl)‐5‐(3‐carboxymethoxyphenyl)‐2‐(4‐sulfophenyl)‐2H‐tetrazolium] experiments, the doses of TXL were determined to be 200, 400, and 800 μg/ml in HPMECs.

### 2.4 Mouse pulmonary functions

Mice were anesthetized with 50 mg/kg sodium pentobarbital (Sigma‐Aldrich) via intraperitoneal injection and then tracheostomized and placed in a forced pulmonary maneuver system (Buxco Research Systems). For determination of the lung function of mice, Boyle's low functional residual capacity (FRC) was performed using the Buxco system (Vanoirbeek et al., 2010). The FRC was determined using Boyle's law FRC maneuver; the dynamic compliance (cdyn) was acquired by the quasistatic pressure–volume (PV) maneuver; and the forced vital capacity (FVC), forced expiratory volume (FEV) at 50 ms (FEV_50_), and inspiratory resistances (RI) were recorded with the fast flow volume maneuver. Each maneuver was repeated at least three times. After the experiment, the mice were deeply anesthetized and decapitated.

### 2.5 Lung microvascular permeability

Lung microvascular permeability was determined by the Evans blue dye method (Lv et al., 2020). Briefly, 50 μl of Evans blue dye (500 μg/50 μl phosphate‐buffered saline [PBS]) per 20 mg/kg body weight was injected intravenously (iv, dorsal tail vein). Two hours after the Evans blue injection, the animals were anesthetized with sodium pentobarbital and perfused with 10 ml of 0.9% sodium chloride solution to remove the blood. Then, the lung tissue was weighed, incubated, and homogenized with formamide (Sigma‐Aldrich) for 48 h at 37°C. The concentration of the dye (μg/mg tissue) was determined in the supernatant using a spectrophotometer at 620 nm against a standard curve.

### 2.6 Tissue collection and morphological analysis

At the end of pulmonary function experiments, mice were sacrificed under deep anesthesia and decapitated to ensure death, and tissues were collected immediately. The mouse left lung without lavage and the total length of aortic arch were fixed in 4% phosphate‐buffered paraformaldehyde (pH 7.4), embedded in paraffin, and sliced into 8‐μm‐thick sections that were stained with hematoxylin and eosin (HE) solution. Alveolar enlargement was determined by the average linear intercept and the ratio of the total length of alveoli to the number of alveoli per field. Histological analysis was performed in 10 visual fields per mouse, and the average value of each mouse was calculated.

For the quantification of atherosclerotic lesions, Oil Red O‐positive lesion surface areas on en face preparations of the whole aorta were measured. Briefly, the aorta from the root to the abdominal area was dissected and fixed with formalin, followed by a careful removal of the connective tissues and longitudinal opening with the intima toward the outside. Then, the tissues were stained in prewarmed Oil Red O solution for 10 min and differentiated in 85% propylene glycol for 5 min at room temperature. The extent of atherosclerotic lesion development was defined as the percentage of the total Oil Red O‐positive lesion area over the total surface area.

For detection of vascular endothelial cadherin (VE‐cadherin; anti‐VE‐cadherin, sc‐9989, 1:50), frozen sections were stained with 8 μM dihydroethidium (DHE; Wako) for 30 min, followed by counterstaining and sealing with mounting medium and antifade reagent (with DAPI [4′,6‐diamidino‐2‐phenylindole; Solarbio]). Fluorescence images were captured under a Zeiss confocal microscope.

### 2.7 Transmission electron microscopy

Fresh left upper lung tissues (1 × 1 × 1 mm) were collected for electron microscopy. The specimen was fixed in 2.5% glutaraldehyde (Sigma‐Aldrich) and phosphate buffer. The specimen was then rinsed in phosphate buffer and postfixed with 1% osmic tetroxide (Sigma‐Aldrich) in phosphate buffer. After graded dehydration in ethyl alcohol and propylene oxide, the specimen was embedded in Spurr resin. Then, the embedded tissue was thin‐sectioned, mounted on copper grids, and stained with uranyl acetate and lead citrate. Images were taken by electron microscopy (H‐7700; Hitachi).

### 2.8 Serum lipid analysis

Mice were fasted overnight, blood was taken from the retrobulbar venous plexus under deep anesthesia, and mice were sacrificed by decapitation. Serum total cholesterol (TC), triglyceride (TG), high‐density lipoprotein (HDL), and low‐density lipoprotein (LDL) levels were determined using enzymatic kits from Jiuqiang Biotechnology Co., Ltd. according to the manufacturer's protocols.

### 2.9 Cell culture and treatment

Human PMECs (human pulmonary microvascular endothelial cells [HPMECs]) were obtained from Yaji Biological. The cells were maintained in DMEM with 10% heat‐inactivated fetal bovine serum (FBS, Gibco) and 1% antibiotic–antimycotic in a humidified incubator under 5% CO_2_ at 37°C. All cells at passages 3–4 were used for the subsequent experiments.

The HPMECs were divided into five groups: (i) control: HPMECs were incubated in regular cell culture conditions; (ii) cigarette smoke extract (CSE): HPMECs were incubated with diluted CSE for 24 h to establish CSE‐induced pulmonary microvascular barrier dysfunction; (iii) CSE + 200 μg/ml TXL: HPMECs were pretreated with TXL (200 μg/ml) for 6 h and incubated with diluted CSE for 24 h; (iv) CSE + 400 μg/ml TXL: HPMECs were pretreated with TXL (400 μg/ml) for 6 h and incubated with diluted CSE for 24 h; (v) CSE + 800 μg/ml TXL: HPMECs were pretreated with TXL (800 μg/ml) for 6 h and incubated with diluted CSE for 24 h. All experiments were performed in triplicate. The dosage of TXL in HPMECs was determined according to the MTS pre‐experiment.

### 2.10 Preparation of CSE

Cigarette smoke extract (CSE) was generated as described previously (Lv et al., 2016). A syringe‐driven apparatus device was designed and operated to allow a stream of smoke to flow into a tube‐shaped trap. The smoke then entered a flask submerged in liquid nitrogen. The amount of smoke obtained was calculated by the increase in the weight inside the flask. The smoke particulates were collected in dimethylsulfoxide (DMSO) at a concentration of 40 mg/ml, filtered through a 0.22‐μm pore filter, and stored at −80°C for subsequent use. Preliminary experiments confirmed that DMSO had no effect on cell proliferation by comparing the DMSO versus no DMSO conditions.

### 2.11 HPMEC monolayer permeability assays

#### 2.11.1 Test of the permeability to FITC–dextran

Cells were grown on Transwell compartments. Tracer experiments with fluorescent dextran were performed using a 12‐well Transwell system (0.4‐μm pore size, 12‐mm diameter, transparent, Costar). HPMECs (1 × 10^5^ cells/well) were plated in Transwell chambers with 500 and 1500 μl culture media in the upper and lower compartments, respectively. The cells were grown for 5 days and replaced with serum‐free medium for 1 h before the treatment. Permeability was measured by the addition of fluorescein isothiocyanate (FITC)–dextran (40 kDa; Sigma‐Aldrich) for 1 h.

#### 2.11.2 Measurement of transendothelial electrical resistance

The cellular barrier properties were assessed by measuring the resistance values across confluent HPMECs using Millicell‐ERS (MERS00002, Millipore; Monaghan‐Benson & Wittchen, 2011). The 12‐well Transwell system was inserted into the 12‐well plate. HPMECs were plated in Transwell chambers. The electrode tips were inserted into the upper and lower compartments, and the resistance (*R* sample) was measured. The value for the blank well represented the blank resistance (*R* blank). The transendothelial electrical resistance (TEER) value was calculated based on the resistance per unit area as (*R* sample − *R* blank) × 1.33 cm (the area of a 12‐well Transwell chamber is 1.33 cm^2^; Tominaga et al., 2015).

### 2.12 Immunofluorescence staining

After pretreated HPMECs reached 70% confluency, the cells were washed with PBS, fixed in 4% paraformaldehyde for 15 min, permeabilized for 5 min with buffer containing 0.1% Triton X‐100, and blocked with goat serum for 1 h at room temperature. The cells were further incubated with primary antibodies (VE‐cadherin, 1:200, SC9989; β‐catenin, 1:200, ab32572) at 4°C overnight. After washing, the cells were incubated with secondary antibodies (goat anti‐mouse, SA00013‐3, 1:200; goat anti‐rabbit SA00013‐2, 1:200). Finally, the cell nuclei were stained using DAPI for an additional 15 min and visualized under a Zeiss confocal microscope (Li et al., 2018).

### 2.13 Enzyme‐linked immunosorbent assays for inflammatory cytokines

Lungs were homogenized in a saline solution (0.9% NaCl), and the mouse serum and the cell culture supernatants were collected via centrifugation (159.84 *g*, 10 min, 4°C) to remove cell debris and stored at −80°C until subsequent analyses. The levels of inflammatory cytokines were quantified using enzyme‐linked immunosorbent assays (ELISAs). The kits were purchased from Proteintech (mouse IL‐1β [interleukin 1 beta] ELISA kit, cat. no. KE10003; Mouse IFN‐γ [interferon gamma] ELISA kit, cat. no. KE10001; Mouse TNF‐α [tumor necrosis factor alpha] ELISA kit, cat. no. KE10002; Human IL‐6 ELISA kit, cat. no. KE00139; Human IL‐1β ELISA kit, cat. no. KE20005) and Zcibio (Human hsCRP [high‐sensitivity C‐reactive protein] ELISA kit, cat. no. ZC‐M6088). The operation steps were strictly carried out according to the instructions of the manufacturer.

### 2.14 Real‐time quantitative polymerase chain reaction

Total RNA was extracted from mouse pulmonary and aortic tissue and HPMECs by an Eastep Super Total RNA extraction kit and measured by a NanoDrop 2000 system (Thermo Scientific). Subsequently, reverse transcription was performed using a Prime‐Script^TM^ RT Reagent kit according to the manufacturer's instructions. Quantitative PCR was carried out on a 7900HT Fast Real‐Time PCR System (Applied Biosystems). Glyceraldehyde 3‐phosphate dehydrogenase (GAPDH) was used as an internal reference, and the primer sequences are shown in Table 2.

**TABLE 2:** Primer sequences used for real‐time quantitative polymerase chain reaction

Genes	Forward (5′ → 3′)	Reverse (5′ → 3′)
VE‐cadherin (mouse)	TGACAACACCGCCAACATC	TGACCAACTGCTCGTGAATC
β‐catenin (mouse)	CCATCTGTGCTCTTCGTCATC	ACTGCTGCTGCGTTCCA
IL‐1β (mouse)	ATCTCGCAGCAGCACATCA	CCAGCACGGTTATCATCATCATCC
TNF‐α (mouse)	AAGCAAGCAGCCAACCAG	CCACAAGCAGGAATGAGAAGA
VCAM‐1 (mouse)	CCAGGACTCAGAATGACTTC AG	CGACCATCTTCACAGGCATT
ICAM‐1 (mouse)	CAGTGAGGAGGTGAATGTATAAGTT	AGGATGTGGAGGAGCAGAGA
GAPDH (mouse)	GGTGAAGGTCGGTGTGAACG	CTCGCTCCTGGAAGATGGTG
VE‐cadherin (human)	GATGTGTCGTGCTCAACTCAT	CCAGTCGTTAAGGAAGTCGTA
β‐catenin (human)	CCGACACCAAGAAGAAGCA GAGAT	GCACGAACAAGCAACTGAACT A
Claudin 5 (human)	TGGTAAGGCTGGTGAGGTC	GGAGTGGAGTATGGAGTTGGA
VCAM‐1 (human)	CTTAAAATGCCTGGGAAGAT GGT	GTCAATGAGACGGAGTCACCA AT
ICAM‐1 (human)	TCATTGACTTGCAGCACCAC AGG	TGCACAGGTAAGAGTGTTCGT TCC
GAPDH (human)	CGACCATTTGTCAAGCTCA	AGGGGTCTACATGGCAACT

Abbreviations: GAPDH, glyceraldehyde 3‐phosphate dehydrogenase; ICAM‐1, intercellular adhesion molecule 1; IL‐1β, interleukin 1 beta; TNF‐α, tumor necrosis factor alpha; VCAM‐1, vascular cell adhesion molecule 1; VE, vascular endothelial.

### 2.15 Western blotting analysis

The protein concentration of mouse lung tissue and HPMECs was determined using a bicinchoninic acid (BCA) protein assay kit before sodium dodecyl sulfate‐polyacrylamide gel electrophoresis (SDS‐PAGE; GenScript). Then, the proteins were transferred to nitrocellulose blotting membranes (Life Sciences).

After the membranes were blocked with Odyssey® Blocking Buffer (Li‐Cor) for 1 h at 37°C, they were incubated with primary antibodies (VE‐cadherin, SC9989; β‐catenin, ab32572; Claudin 5, ab131259; nuclear factor kappa B [NF‐қB], CST8242s, MA, USA; β‐actin, GB11001, Servicebio) at 4°C overnight, followed by incubation with secondary antibodies at 37°C for 1 h. Finally, the membranes were rinsed with Tris‐buffered saline with Tween 20 (TBST) before scanning by an Odyssey two‐color infrared imaging system (Li‐Cor). β‐Actin was used as an internal control.

### 2.16 Statistical analysis

Statistical significance of differences was assessed using one‐way analysis of variance (ANOVA) or repeated‐measures analysis, followed by post hoc analysis using Fisher's least significant difference (LSD) multiple comparison test. Differences at *p* < .05 were regarded as statistically significant. Data are presented as the mean ± SEM.

## 3 RESULTS

### 3.1 Cigarette smoke aggravates the high‐fat diet‐induced lung function decline in ApoE ^−/−^ mice, and the TXL–Ato combination improves lung function in ApoE ^−/−^ mice treated with cigarette smoke combined with a high‐fat diet

**FIGURE 1:**
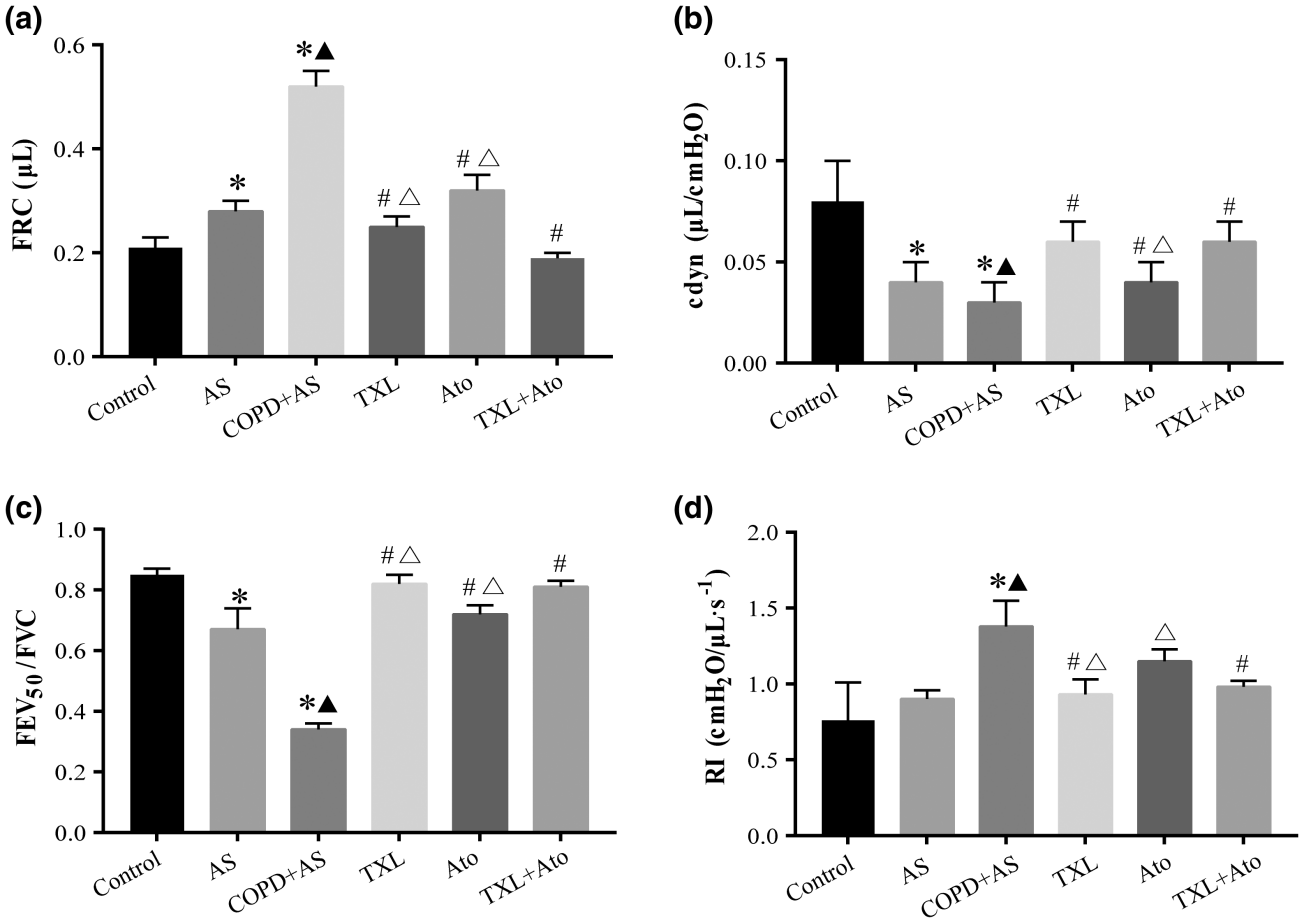
Lung function tests' data revealed that Tongxinluo–atorvastatin (TXL–Ato) combination improved the lung function of COPD + AS mice. Cigarette smoke extract (CSE) combined with high‐fat diet induced a significant increase in the functional residual capacity (FRC) (a) and inspiratory resistances (RI) (d) and a significant decrease in dynamic compliance (cdyn) (b) and forced expiratory volume (FEV) at 50 ms/forced vital capacity (FEV_50_/FVC) (c) in the COPD + AS group. The TXL–Ato combination improved the lung function, including reduced FRC, RI, and preserved cdyn and FEV_50_/FVC compared to the COPD + AS group, *n* = 8, *p < .05 versus control group; ^▲^ *p* < .05 versus AS group; ^#^ *p* < .05 versus COPD + AS group; ^△^ *p* < .05 versus TXL + Ato group.

To ensure that cigarette smoke treatment of ApoE^−/−^ mice using our apparatus can reproduce the pathologic features of COPD, we assessed their lung functions by a forced pulmonary maneuver system. The results showed that cigarette smoke exposure aggravates the emphysema‐like phenotype (FRC and cdyn) and the airflow‐limited phenotype (FEV_50_/FVC and RI) induced by a high‐fat diet in mice. A significant increase in FRC (Figure 1a) and a significant decrease in cdyn (Figure 1b) and FEV_50_/FVC (Figure 1c) were observed in the AS and COPD + AS groups compared to the control group. In particular, a significant increase was noted in FRC and RI (Figure 1d), and a significant decrease in cdyn and FEV_50_/FVC was observed in the COPD + AS group compared to the AS group. Compared to those of the COPD + AS group, FRC and RI decreased significantly and FEV_50_/FVC and cdyn increased significantly in the TXL and TXL + Ato groups. A significant decrease in FRC and a significant increase in FEV_50_/FVC and cdyn were observed in the Ato group compared to the COPD + AS group.

Nonetheless, the Ato group showed no significant change in RI compared to the COPD + AS group. A significant decrease was noted in FRC and RI, and a significant increase in FEV_50_/FVC was observed in the TXL + Ato group compared to the TXL group; however, cdyn in the TXL group did not show a significant difference compared to that in the TXL + Ato group. A significant decrease in FRC and RI and a significant increase in FEV_50_/FVC and cdyn were observed in the TXL + Ato group compared with the Ato group.

These data indicated that cigarette smoke exposure reduced lung function induced by a high‐fat diet in mice. The TXL and Ato combination improved lung function compared with either drug alone.

### 3.2 Cigarette smoke aggravates high‐fat diet‐induced pulmonary microvascular endothelial barrier dysfunction in ApoE ^−/−^ mice, and the TXL–Ato combination protects pulmonary microvascular endothelial barrier function in ApoE ^−/−^ mice treated with cigarette smoke combined with a high‐fat diet

**FIGURE 2:**
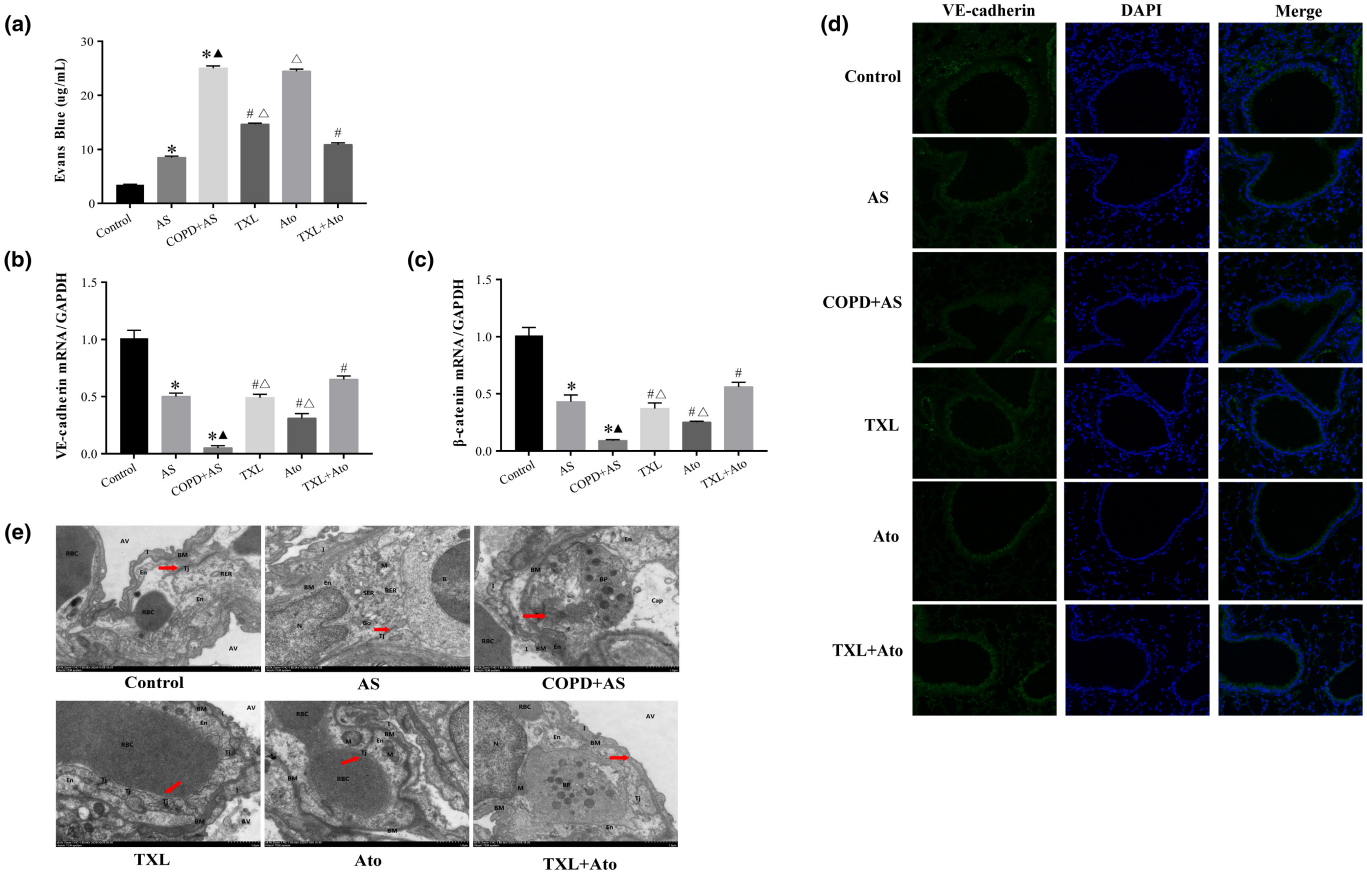
Effects of Tongxinluo–atorvastatin (TXL–Ato) combination on pulmonary microvascular barrier. (a) Evans blue content in lung tissue was measured in each group to assess pulmonary microvascular permeability. Gene expression levels of vascular endothelial cadherin (VE‐cadherin) (b) and β‐catenin (c) were determined by real‐time quantitative polymerase chain reaction (RT‐qPCR). (d) Immunofluorescent analysis of VE‐cadherin (×400). (e) Scale: 1 μm. Transmission electron microscopy (TEM) of the pulmonary microvasculature region, *n* = 3, *p < .05 versus control group; ^▲^ *p* < .05 versus AS group; ^#^ *p* < .05 versus COPD + AS group; ^△^ *p* < .05 versus TXL + Ato group.

Evans blue content in the lung tissue was measured in each group to assess pulmonary microvascular permeability, which plays a pivotal role in the pulmonary inflammatory response and acts as a therapeutic target for the treatment of COPD. To address whether the TXL–Ato combination modulates pulmonary microvascular permeability in vivo, we assessed pulmonary microvascular leakage. In addition, transmission electron microscopy (TEM) was used to examine the ultrastructural changes in lung tissues, vascular endothelial cadherin (VE‐cadherin) and β‐catenin levels were determined by real‐time quantitative polymerase chain reaction (RT‐qPCR), and the expression of VE‐cadherin was determined by immunofluorescence staining. The results showed an increase in Evans blue content in the lung tissue (Figure 2a) in the AS and COPD + AS groups compared to the control group and a significant increase in Evans blue content in the COPD + AS group compared to the AS group. Compared to the COPD + AS group, the TXL group and TXL + Ato group had significantly decreased Evans blue contents in the lung tissues, but the Ato group showed no change. However, the TXL–Ato combination significantly decreased the Evans blue content compared to those of the TXL and Ato groups. The messenger RNA (mRNA) levels of VE‐cadherin and β‐catenin (Figure 2b,c) in mice were significantly reduced in the AS and COPD + AS groups compared to the control group. Additionally, a significant decrease in VE‐cadherin and β‐catenin expression was detected in the COPD + AS group compared to the AS group. However, VE‐cadherin and β‐catenin mRNA expression was significantly increased in the TXL, Ato, and TXL–Ato groups compared to the COPD + AS group. Compared to those in the TXL group, the mRNA levels of VE‐cadherin and β‐catenin were significantly increased in the TXL–Ato group. Compared to those of the Ato group, the mRNA levels of VE‐cadherin and β‐catenin were significantly increased in the TXL–Ato group. Immunofluorescence staining of VE‐cadherin in lung tissues demonstrated that the COPD + AS group had decreased expression of VE‐cadherin, while TXL–Ato combination treatment restored VE‐cadherin levels at cell–cell junctions (Figure 2d).

Type I epithelial cell cytoplasm (I), vascular basement membrane (BM), and endothelial cell cytoplasm (En) together form the gas–blood barrier. The ultrastructure was observed by transmission electron microscopy (TEM), and the endothelial cells lining the lumen of the pulmonary microvasculature and the Tj proteins between adjacent endothelial cells maintained the integrity of the pulmonary barrier (Figure 2e). In the control group, the structure of the air–blood barrier was complete, the endothelial cells showed slight edema, the Tjs were dense, and the BM was smooth. The capillary En of the COPD + AS group had moderate edema, the cell membrane was incomplete, the Tjs between the cells were blurred and nearly disappeared, and the BM was blurred and discontinuous. The BM in the air–blood barrier of the TXL group was fuzzy and discontinuous, and the Tjs were fuzzy. The air–blood barrier in the Ato group showed edema, the BM was blurred, and the Tjs partially disappeared. The air–blood barrier damage in the TXL + Ato group was slightly weaker than that in the TXL group, and the Tjs were also slightly better than those in the TXL group.

These data indicated that cigarette smoke aggravates high‐fat diet‐induced pulmonary microvascular endothelial barrier dysfunction in ApoE^−/−^ mice. The TXL–Ato combination significantly attenuated pulmonary microvascular permeability under cigarette smoke combined with a high‐fat diet. In the treatment of COPD, TXL may have more targets than Ato, such as the protective effect of the pulmonary microvascular barrier.

### 3.3 Cigarette smoke aggravates high‐fat diet‐induced pulmonary inflammation in ApoE ^−/−^ mice, and the TXL–Ato combination inhibits pulmonary inflammation in ApoE ^−/−^ mice treated with cigarette smoke combined with a high‐fat diet

**FIGURE 3:**
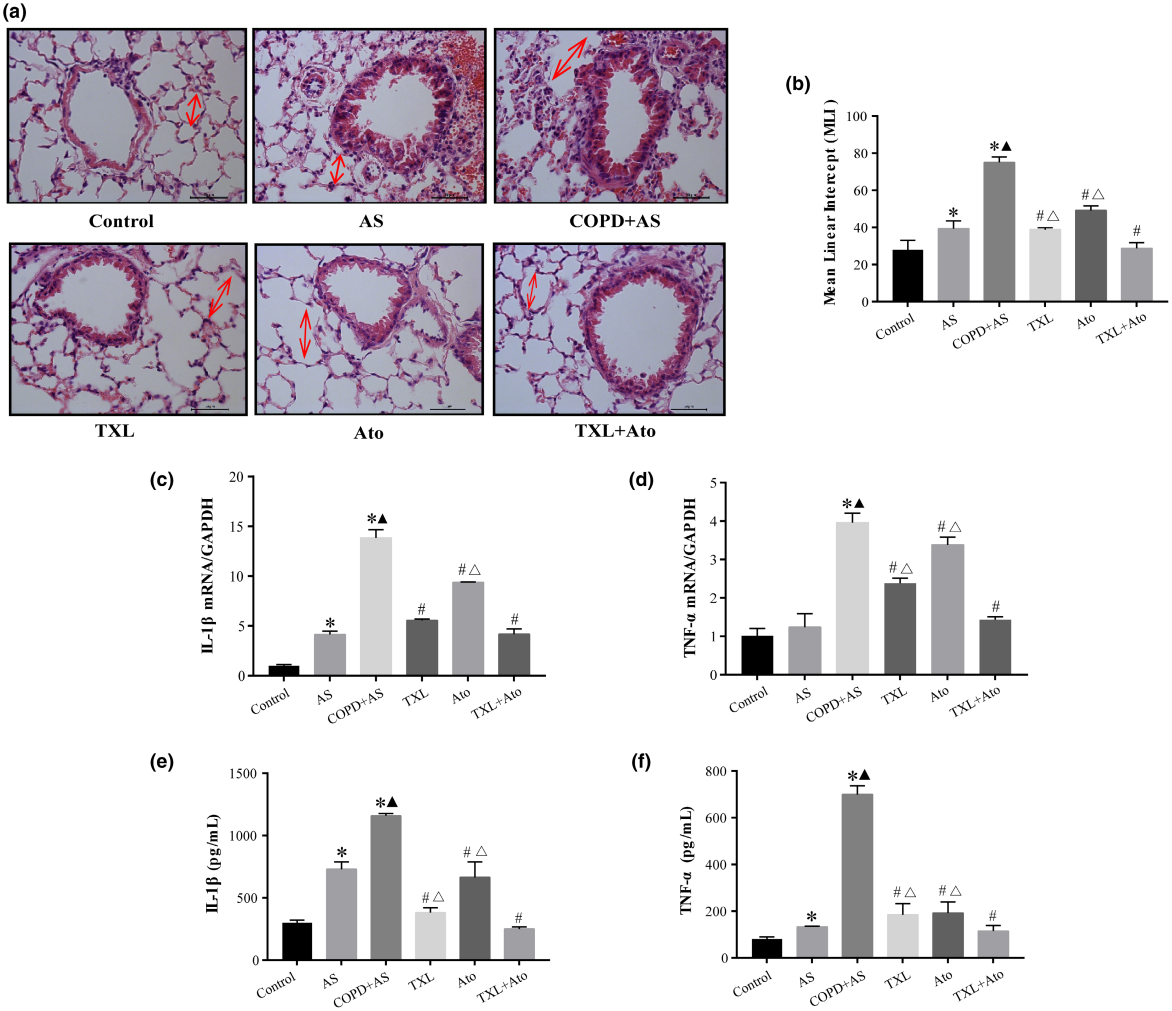
Effects of Tongxinluo–atorvastatin (TXL–Ato) combination on pulmonary inflammation of COPD + AS mice, (a) Scale: 50 μm. Lung tissue morphology was examined by hematoxylin and eosin (HE) staining. (b) Image‐Pro Plus was performed to measure the mean linear intercept (MLI). Each plot represents mean ± SEM from seven different animals. The expression of interleukin 1β (IL‐1β) (c) and tumor necrosis factor alpha (TNF‐α) (d) levels in lung tissues was determined by real‐time quantitative polymerase chain reaction (RT‐qPCR). The IL‐1β (e) and TNF‐α (f) levels in the supernatant of lung homogenates were measured according to enzyme‐linked immunosorbent assay (ELISA). *n* = 6, *p < .05 versus Control group; ^▲^ *p* < .05 versus AS group; ^#^ *p* < .05 versus COPD + AS group; ^△^ *p* < .05 versus TXL + Ato group.

Lung histopathology, the gene expression level in lung tissues, and the supernatant of lung homogenates were used to further assess airway inflammation and airway remodeling. In the normal lung tissue, the contour of the bronchial tube was clear, and only a slight inflammatory cell infiltration was observed around the bronchial submucosa. Perivascular and peribronchial inflammatory cell infiltration was observed in the AS group. Compared to the AS group, the COPD + AS group showed profound peribronchial and/or perivascular inflammatory infiltration and mucus secretion. The TXL and Ato groups had perivascular and peribronchial inflammatory infiltration and mild inflammation in the alveolar septa, predominantly composed of mononuclear cells. The TXL + Ato group showed the presence of few inflammatory cells in the peribronchial space and mild infiltration in the alveolar septa (Figure 3a).

Mean linear intercept (MLI) analysis also suggested an enlargement of alveoli in the COPD + AS group (Figure 3b) compared to the AS group. Compared to that in the COPD + AS group, the MLI in the three treatment groups was significantly decreased, and the MLI in the TXL + Ato group was significantly decreased compared to those in the TXL and Ato groups. The expression of inflammatory factors in mouse lung tissue was measured using RT‐qPCR (Figure 3c,d), and IL‐1β and TNF‐α expression was significantly elevated in the COPD + AS group compared to the AS group. Furthermore, the TXL–Ato combination significantly decreased the expression of TNF‐α compared to those in the TXL and Ato groups. However, the TXL–Ato group showed a significant decrease in IL‐1β mRNA levels compared to the Ato group, but there was no significant difference in the TXL group. ELISA results showed that (Figure 3e,f) the IL‐1β and TNF‐α levels in the COPD + AS group significantly increased compared to those in the AS group, and the TXL + Ato group showed significantly decreased IL‐1β and TNF‐α levels compared to the TXL and Ato groups.

These data together with lung function data suggested that cigarette smoke aggravates high‐fat diet‐induced pulmonary inflammation in ApoE^−/−^ mice. The TXL–Ato combination inhibited pulmonary inflammation in ApoE^−/−^ mice treated with cigarette smoke combined with a high‐fat diet, and the TXL–Ato combination exerted a better anti‐inflammatory effect than TXL and Ato.

### 3.4 Cigarette smoke aggravates high‐fat diet‐induced systemic inflammation in ApoE ^−/−^ mice, and the TXL–Ato combination attenuates systemic inflammation in ApoE ^−/−^ mice treated with cigarette smoke combined with a high‐fat diet

**FIGURE 4:**
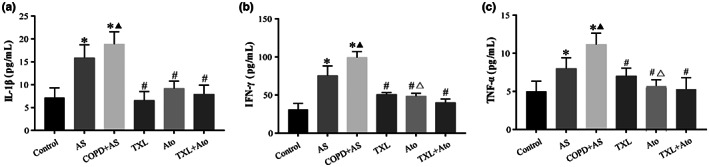
Inflammatory factors (interleukin 1β [IL‐1β], interferon gamma [IFN‐γ] and tumor necrosis factor alpha [TNF‐α]) in serum were detected by enzyme‐linked assay (ELISA). Cigarette smoke exposure combined with high‐fat diet induced a significant increase in the levels of IL‐1β (a), IFN‐γ (b), and TNF‐α (c) in the COPD + AS group, and Tongxinluo–atorvastatin (TXL–Ato) combination decreased the inflammation level compared to that in the COPD + AS group, *n* = 6, *p < .05 versus control group; ^▲^ *p* < .05 versus AS group; ^#^ *p* < .05 versus COPD + AS group; ^△^ *p* < .05 versus TXL + Ato group.

To test the potential effect of cigarette smoke combined with a high‐fat diet on circulatory inflammation, we assessed the levels of different cytokines in the serum of the animal model. Figure 4 shows significantly increased levels of IL‐1β (Figure 4a), IFN‐γ (Figure 4b), and TNF‐α (Figure 4c) in the COPD + AS group compared to the AS group. TXL, Ato, and the TXL–Ato combination significantly decreased the expression of IL‐1β, IFN‐γ, and TNF‐α compared to that in the COPD + AS group, while the TXL–Ato combination significantly decreased the expression of IFN‐γ and TNF‐α compared to that in the Ato group. However, there was no significant difference in IL‐1β in the TXL + Ato group compared with the TXL group and the Ato group.

These findings indicated that with the destruction of pulmonary microvascular barrier function, pulmonary inflammation in COPD aggravates systemic inflammation. The TXL–Ato combination was more effective in reducing circulatory inflammation than either drug alone.

### 3.5 Cigarette smoke aggravates high‐fat diet‐induced AS lesions in ApoE ^−/−^ mice, and the TXL–Ato combination attenuates AS lesions in ApoE ^−/−^ mice treated with cigarette smoke combined with a high‐fat diet

**FIGURE 5:**
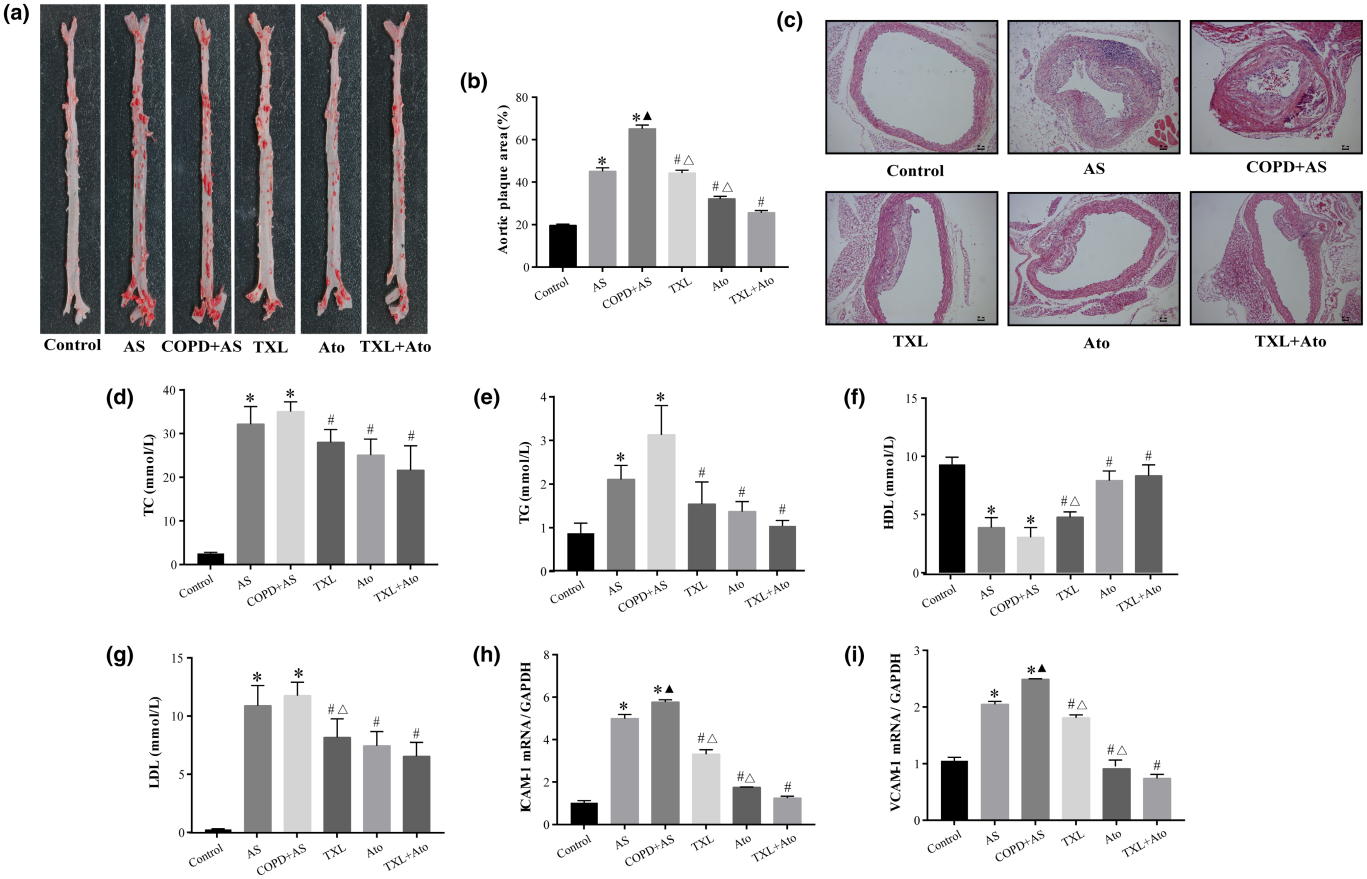
Cigarette smoke aggravates high‐fat diet‐induced atherosclerotic (AS) lesions in AS and COPD + AS groups and Tongxinluo–Ato (TXL–Ato) combination attenuated atherosclerotic lesions compared to the COPD + AS group, (a) Atherosclerotic (AS) lesion shown with Oil Red O staining; (b) Quantitative analysis of plaque areas in the aortic sinus by Image‐Pro Plus software. (c) Scale: 50 μm. Aortic root morphology was examined by hematoxylin and exosin (HE) staining. Blood lipid levels of total cholesterol (TC) (d), triglyceride (TG) (e), high‐density lipoprotein‐C (HDL‐C) (f), and low‐density lipoprotein‐C (LDL‐C) (g) of serum. Real‐time quantitative polymerase chain reaction (RT‐qPCR) determines the messenger RNA (mRNA) levels of endothelial damage biomarkers intercellular adhesion molecule 1 (ICAM‐1) (h) and vascular cell adhesion molecule 1 (VCAM‐1) (i) in the aorta tissue. *n* = 6, *p < .05 versus control group; ^▲^ *p* < .05 versus AS group; ^#^ *p* < .05 versus COPD + AS group; ^△^ *p* < .05 versus TXL + Ato group.

Figure 5a,b show that the COPD + AS group had significantly increased formation of atherosclerotic plaques compared to the AS group. The plaques showed cholesterol‐rich lipid clefts within the intimal layer containing distinct, large macrophage‐derived foam cells (Figure 5c). However, compared to the COPD + AS group, the TXL, Ato, and TXL + Ato groups had significantly decreased formation of atherosclerotic plaques. The TXL–Ato combination significantly decreased atherosclerotic lesions compared to those in the Ato group (Figure 5b).

Compared to those of the control group, the TC, TG, and low‐density lipoprotein C (LDL‐C) levels were significantly increased and the high‐density lipoprotein C (HDL‐C) level was significantly decreased in the COPD + AS and AS groups. However, the levels of TC, TG, LDL‐C, and HDL‐C in the COPD + AS group did not differ significantly from those in the AS group. The administration of TXL, Ato, and the TXL–Ato combination reduced the serum lipid levels compared to those of the COPD + AS group. The TXL–Ato combination significantly increased the HDL‐C level and significantly decreased the LDL level compared to those in the TXL group, and the TC and TG levels did not differ significantly between the TXL + Ato and TXL groups. Compared to those of the Ato group, the TC, TG, LDL‐C, and HDL‐C levels showed no significant differences in the TXL + Ato group (Figure 5d–g).

The RT‐qPCR analysis revealed that vascular cell adhesion molecule 1 (VCAM‐1) and intercellular adhesion molecule 1 (ICAM‐1) mRNA expression (Figure 5h,i) was high in the aortic tissue of the AS and COPD + AS groups compared to the control group. VCAM‐1 and ICAM‐1 mRNA expression was significantly increased in the COPD + AS group compared to the AS group. Nevertheless, the TXL, Ato, and TXL–Ato groups had substantially reduced VCAM‐1 and ICAM‐1 mRNA levels compared to the COPD + AS group. Compared to that of the TXL group, VCAM‐1 and ICAM‐1 mRNA expression was significantly reduced in the TXL + Ato group. Compared to those of the Ato group, the mRNA levels of ICAM‐1 and VCAM‐1 were significantly reduced in the TXL + Ato group.

These data demonstrated that subjecting ApoE^−/−^ mice to cigarette smoke aggravates high‐fat diet‐induced AS lesions and that the combined use of TXL with Ato enhances the antiatherosclerotic effect of Ato.

### 3.6 TXL protects against CSE‐induced pulmonary microvascular endothelial barrier dysfunction in HPMECs

**FIGURE 6:**
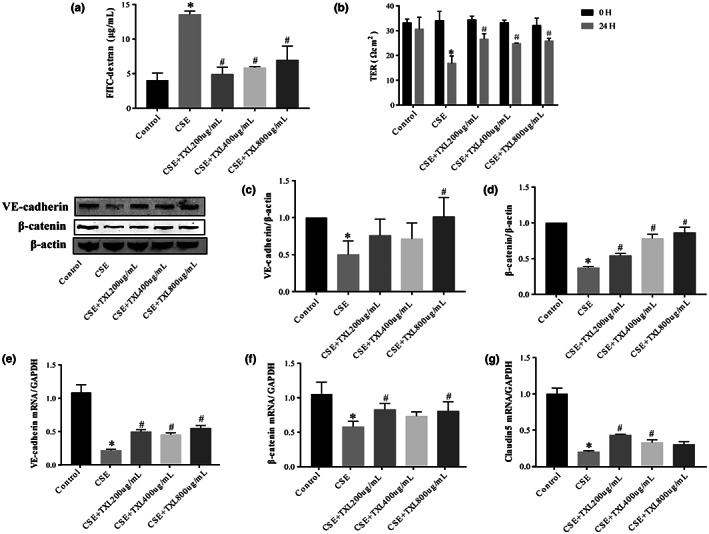
Tongxinluo (TXL) protects human pulmonary microvascular endothelial cells (HPMECs') pulmonary microvascular endothelial barrier dysfunction induced by cigarette smoke extract (CSE). The permeability of the endothelial monolayer was tested by measuring the influx of fluorescein isothiocyanate–dextran (FITC–dextran) (a) and the transendothelial electrical resistance (TEER) (b). The expression of Tj proteins vascular endothelial cadherin (VE‐cadherin) (c) and β‐catenin (d) were detected in HPMECs using Western blot. The expression of Tj proteins' messenger RNA (mRNA) levels of VE‐cadherin (e), β‐catenin (f), and Claudin 5 (g) was detected by real‐time quantitative polymerase chain reaction (RT‐qPCR). *n* = 3, *p < .05 versus Control group; ^#^ *p* < .05 versus CSE group.

**FIGURE 7:**
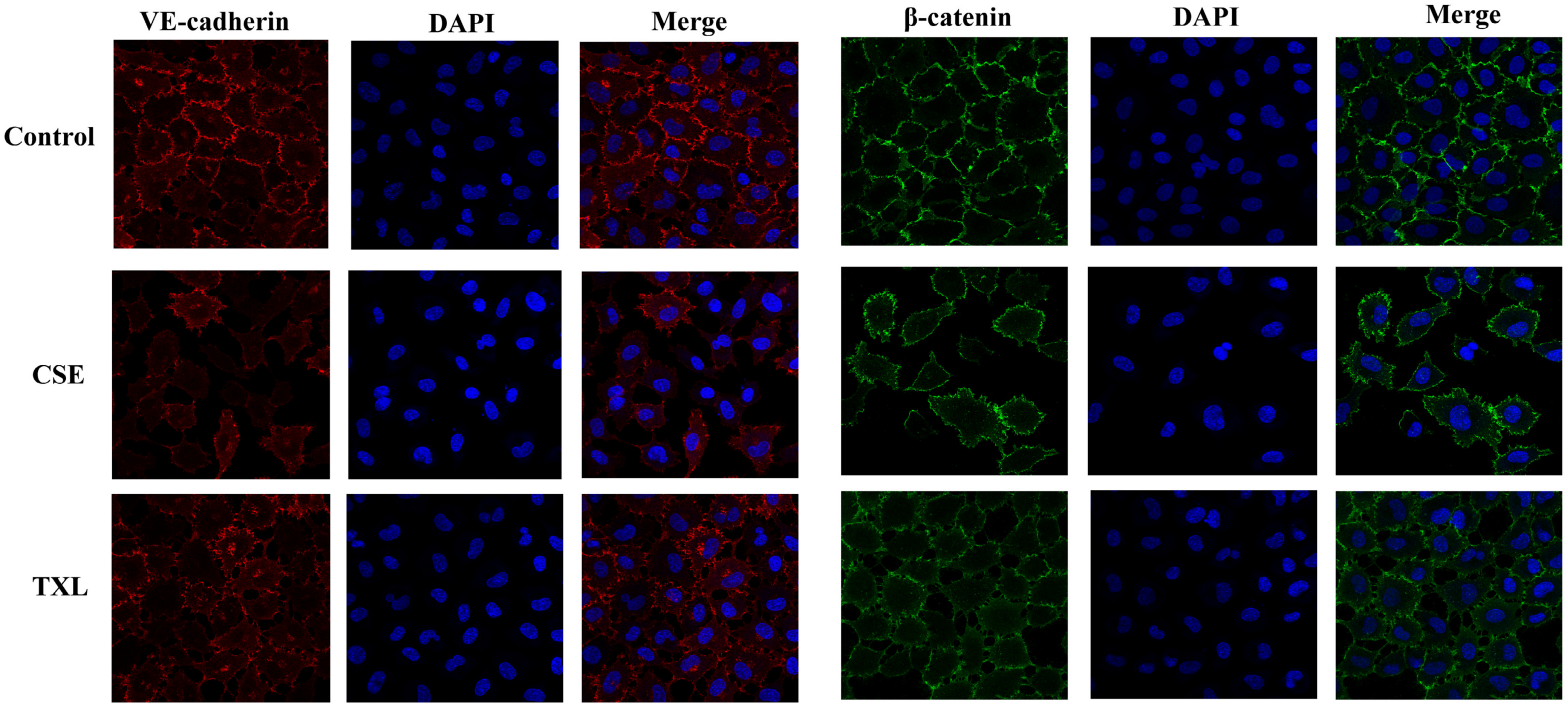
Tight junction (Tj) proteins (vascular endothelial cadherin [VE‐cadherin] and β‐catenin) were evaluated by immunofluorescence microscopy (×400).

Herein, we demonstrated that pulmonary microvascular barrier dysfunction is critical in COPD exacerbation of AS in vivo. Next, we verified whether the key mechanism by which COPD aggravates AS is pulmonary microvascular barrier dysfunction through in vitro experiments and whether TXL can protect the function of the pulmonary microvascular barrier. CSE was applied to HPMECs for 24 h to mimic pulmonary microvascular endothelial barrier dysfunction in vitro. The permeability of the endothelial monolayer was tested by measuring the influx of FITC–dextran and the TER across cells. CSE induced endothelial barrier dysfunction, as shown by increased FITC–dextran leakage (Figure 6a) and decreased TER (Figure 6b). The influx of FITC–dextran was significantly decreased, and the TER across cells was significantly increased by TXL (Figure 6b). Immunofluorescence staining revealed that CSE downregulated the expression of VE‐cadherin and β‐catenin in HPMECs (Figure 7), which was improved by TXL.

We also tested the changes in the expression of Tj proteins and the relative expression levels of VE‐cadherin and β‐catenin, as determined by Western blots (Figure 6c,d), indicating that CSE significantly decreased VE‐cadherin and β‐catenin protein levels and that TXL significantly increased the β‐catenin protein level. TXL at 800 μg/ml upregulated the level of VE‐cadherin; however, 400 and 200 μg/ml TXL did not show any significant effect on VE‐cadherin. Similarly, the RT‐qPCR (Figure 6e–g) results showed that the VE‐cadherin, β‐catenin, and Claudin‐5 mRNA levels were downregulated in the CSE group, and TXL reversed this effect.

The effects of TXL against pulmonary microvascular barrier dysfunction induced by CSE were consistent with the finding that TXL attenuates pulmonary microvascular hyperpermeability under cigarette smoke combined with a high‐fat diet in vivo.

### 3.7 TXL protects against CSE‐induced HPMEC inflammation

**FIGURE 8:**
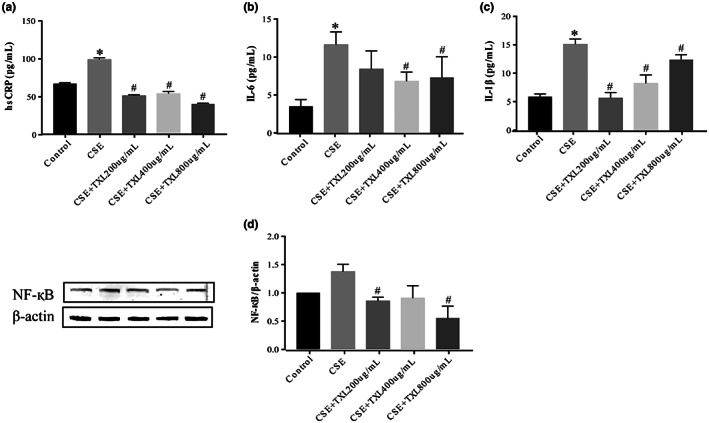
Tongxinluo (TXL) against cigarette smoke extract (CSE)‐induced inflammation in human pulmonary microvascular endothelial cells (HPMECs). The cell supernatants were assayed for high sensitivity C‐reactive protein (hsCRP) (a), interleukin 6 (IL‐6) (b), and interleukin 1β (IL‐1β) (c) by enzyme‐linked immunosorbent assay (ELISA). (d) Nuclear factor‐kappa B (NF‐κB) protein level was detected by Western blot, *n* = 3, *p < .05 versus control group; ^#^ *p* < .05 versus CSE group.

Next, we investigated the correlation between inflammatory signaling and increased permeability of the pulmonary microvascular barrier induced by CSE. ELISAs revealed that CSE exposure elevated the levels of IL‐6, IL‐1β, and high sensitivity C‐reactive protein (hsCRP) in the media supernatant of the cells (Figure 8a–c), while TXL inhibited the levels of IL‐6, IL‐1β, and hsCRP. Western blots were used to analyze the NF‐қB level (Figure 8d). The protein expression of NF‐қB was increased in the CSE group compared to the control group, and 200 and 800 μg/ml TXL significantly reduced the level of NF‐қB compared to that in the CSE group. However, 400 μg/ml TXL did not exert a significant effect on NF‐қB compared to that in the CSE group. Collectively, TXL suppressed CSE‐mediated inflammation, which was consistent with the results of TXL‐inhibited systemic inflammation in the ApoE^−/−^ mice treated with cigarette smoke combined with a high‐fat diet.

### 3.8 TXL protects HPMECs from endothelial damage

**FIGURE 9:**
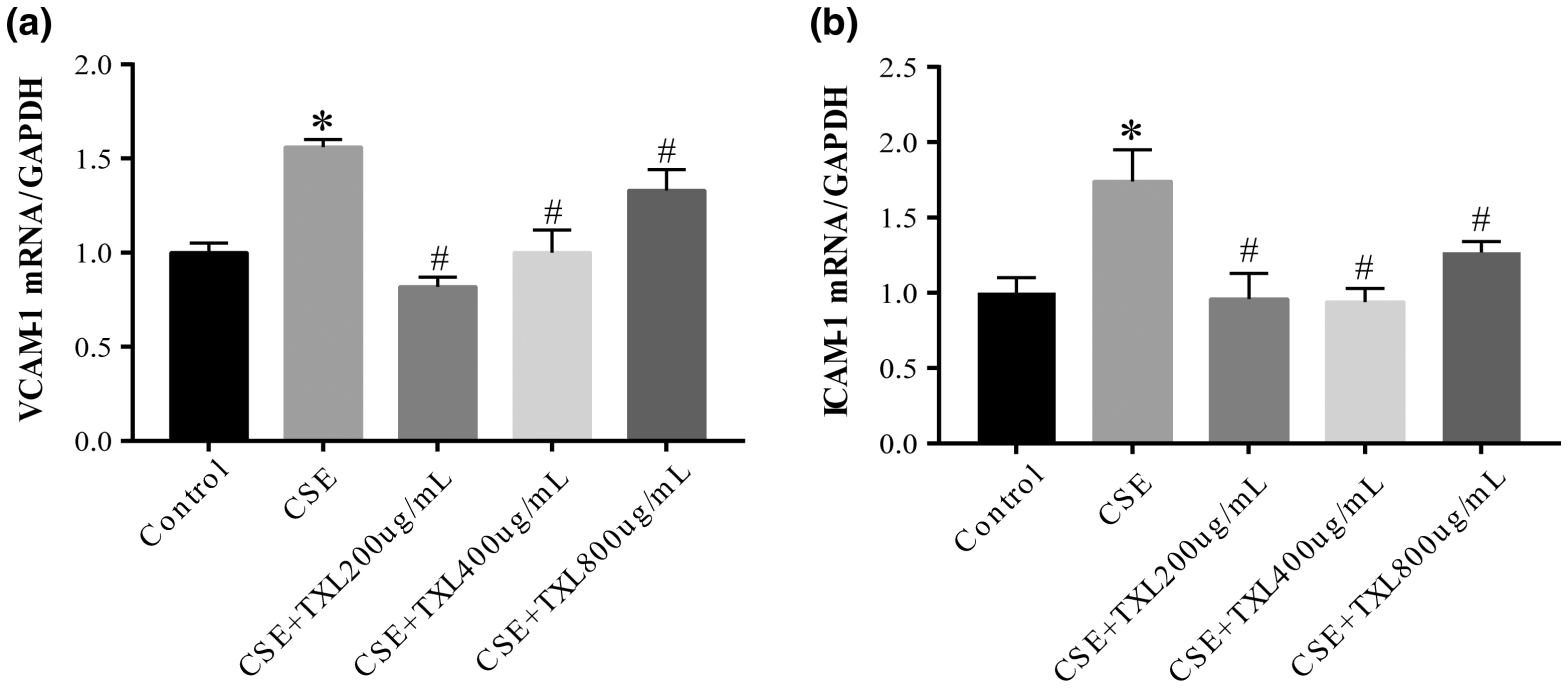
Tongxinluo (TXL) protects human pulmonary microvascular endothelial cells (HPMECs) from endothelial damage. Real‐time quantitative polymerase chain reaction (RT‐qPCR) assay for endothelial damage biomarkers intercellular adhesion molecule 1 (ICAM‐1) (a) and vascular cell adhesion molecule 1 (VCAM‐l) (b) messenger RNA (mRNA) levels in HPMECs. ICAM‐1 and VCAM‐1 mRNAs were significantly upregulated after HPMECs' exposure to cigarette smoke extract (CSE), and pretreatment with TXL significantly counteracted CSE‐induced ICAM‐1 and VCAM‐1 mRNA upregulation in HPMECs. *n* = 3, *p < .05 versus control group; ^#^ *p* < .05 versus CSE group.

A previous study demonstrated that CSE mediates endothelial damage under various insults. Furthermore, TXL inhibits endothelial damage in HPMECs. Therefore, the present study evaluated the effects of TXL on the mRNA expression of ICAM‐1 and VCAM‐1. The current results showed that the expression of these mRNAs was significantly upregulated after HPMECs were exposed to CSE, and pretreatment with TXL significantly counteracted this CSE‐induced effect (Figure 9a,b).

## 4 DISCUSSION

The present study elucidated the effects of TXL on COPD combined with AS in vivo and in vitro. Several observations are summarized as follows: (i) The key mechanism of COPD exacerbation of AS is pulmonary microvascular barrier dysfunction, which leads to pulmonary inflammation triggering systemic inflammation, thus exacerbating the severity of AS. (ii) TXL enhances the antiatherosclerotic effect of Ato by protecting pulmonary microvascular barrier function in mice with COPD combined with AS. (iii) TXL suppresses CSE‐induced pulmonary microvascular barrier dysfunction and inflammation in HPMECs.

According to the Global Burden of Disease report, approximately 3.2 million people died of COPD in 2017 (Li et al., 2020), accounting for 81.7% of all deaths from chronic respiratory diseases. CVD is the leading cause of death in patients with COPD, accounting for 25%–27% of patients with mild to moderate COPD (Calverley et al., 2007; Mannino et al., 2006), and the two diseases are often combined (Vestbo et al., 2013). COPD is known to be an independent risk factor contributing to the development of atherosclerotic vascular disease (Morgan et al., 2018). Some studies have demonstrated that in addition to inflammation in the lungs, COPD patients experience a significant increase in systemic inflammatory markers (CRP, IL‐6, and TNF‐α; Shaw et al., 2014), which can interact with endothelial cells and deposit locally in AS lesions, inducing the expression of adhesion factors and chemokines in endothelial cells and promoting their dysfunction and the formation of atherosclerotic plaques (Eickhoff et al., 2008). It has been suggested that the potential mechanism of COPD combined with AS is systemic inflammation (Morgan et al., 2018). Smoking may lead to the secretion of inflammatory mediators (such as IL‐6, TNF‐α, and IL‐1β) in the peripheral airway of individuals with COPD, thus leading to increased airway microvascular permeability (Chen, Li, Qi, et al., 2018). The early occurrence of microvascular disease may be the driving factor for the development of COPD chronic airway inflammatory disease. PMVECs are one of the most abundant cells in lung tissue and form the innermost layer of pulmonary microcirculation exchange vessels through intercellular junction interactions and connections with the extracellular matrix. The functional and structural integrity of PMVECs is crucial for maintaining normal lung physiological functions (Darwish & Liles, 2013). In pulmonary inflammation, PMVECs are one of the first effector cells attacked by inflammatory factors. The destruction of the structural and functional barriers maintained by PMVECs and the resulting increase in cell permeability underlie the pathology of various pulmonary diseases (Scott et al., 2013). PMVECs act under the action of inflammatory factors; endothelial cells cause edema. However, many inflammatory cells are “impounded” in the lung, and the aggregated inflammatory cells are activated and release many inflammatory mediators. These inflammatory mediators can directly damage microvascular endothelial cells and activate more inflammatory cells, which together trigger a cascade of inflammatory reactions and systemic inflammation (Li et al., 2015). Pulmonary microvascular barrier dysfunction is caused by pulmonary inflammation that stimulates the apoptosis of PMVECs or changes in recombinant endothelial cytoskeleton and intercellular junction proteins, resulting in abnormal cell structure and function, impaired cell barrier function, and increased cell monolayer permeability (Wang et al., 2012). Thus, we hypothesized that pulmonary microvascular barrier dysfunction, followed by pulmonary inflammation leading to systemic inflammation, is the potential mechanism by which COPD aggravates AS.

Smoking is a common risk factor for both COPD and AS. Previous studies have shown that cigarette smoke causes systemic inflammation in mice, similar to that observed in COPD patients with AS. In this study, ApoE^−/−^ mice exposed to cigarette smoke combined with a high‐fat diet for 20 weeks were used to establish a model of COPD combined with AS to explore the role of pulmonary microvascular barrier dysfunction in pulmonary inflammation leading to systemic inflammation, which results in COPD aggravation of AS. We observed that peripheral airway inflammatory mediators (IL‐1β and TNF‐a) and lung tissue pathology were more severe in the ApoE^−/−^ mice induced by smoking combined with a high‐fat diet than in the mice induced by a high‐fat diet alone. In addition, smoking aggravated the decline in lung function, including increased pulmonary resistance (FRC and RI) and a reduction in elastic recoil (cdyn and FEV_50_/FVC), in the mice induced by a high‐fat diet. The alterations in lung function observed in this study were similar to changes in lung function in humans with emphysema. These data were consistent with the findings of the study by Bezerra et al. (Bezerra et al., 2011), in which mice were exposed to the same protocol of cigarette smoke. We further observed that serum systemic inflammatory markers (IL‐1β, IFN‐γ, and TNF‐α), aortic histopathology (hematoxylin–eosin and Oil Red O staining), serum lipid levels, and biomarkers of aortic endothelial injury (ICAM‐1 and VCAM‐1) were increased in the mice induced by cigarette smoking combined with a high‐fat diet compared to the mice induced by a high‐fat diet alone. These results indicated that smoking combined with a high‐fat diet not only induced lung inflammation (IL‐1β and TNF‐α) but also aggravated systemic inflammation (IL‐1β, IFN‐γ, and TNF‐α) in the circulation and the degree of AS lesions in mice. This phenomenon was consistent with relevant clinical studies; elevated circulating inflammatory markers (such as CRP, IL‐6, IL‐8, IFN‐γ, TNF‐α, fibrinogen, and white blood cell count) in patients with COPD directly stimulate various early atherosclerotic processes, including increased endothelial cell adhesion molecules (ICAM‐1 and VCAM‐1; Pasceri et al., 2000) and increased uptake of LDL by macrophages (Zwaka et al., 2001). Reportedly, C‐reactive protein (CRP) has a direct effect on endothelial function (Verma et al., 2002, 2004) and promotes the formation of atherosclerotic plaques (Eickhoff et al., 2008). To verify whether pulmonary microvascular barrier dysfunction is closely related to pulmonary inflammation leading to systemic inflammation and then COPD aggravating AS, we further detected relevant indicators of pulmonary microvascular barrier dysfunction. Pulmonary microvascular permeability (Evans blue content, VE‐cadherin, and β‐catenin expression) was observed in vivo. The results showed that the pulmonary microvascular permeability of the mice induced by smoking combined with a high‐fat diet was significantly increased, and the expression of VE‐cadherin and β‐catenin in lung tissue was significantly decreased compared to that in the mice induced by a high‐fat diet. We further verified the results through in vitro experiments. TER and macromolecular permeability are known to reflect endothelial cell barrier function. We established a CSE‐induced pulmonary barrier dysfunction model in HPMECs for 24 h in vitro and demonstrated that CSE could increase pulmonary microvascular permeability by decreasing TER and increasing the influx of FITC–dextran into the HPMEC monolayer while reducing the expression of Tj proteins (VE‐cadherin and β‐catenin). NF‐қB drives the inflammatory response by inducing the expression of proinflammatory and antiapoptotic genes (Colarusso et al., 2017). Inflammatory mediators, including CRP, TNF‐α, IL‐6, and VCAM‐1, are produced by various cells in response to stimulation by conserved tissue damage signals (Netea et al., 2017). These mediators contribute to the development of chronic inflammation and a proatherogenic lipid profile (Iqbal et al., 2017; Tamakoshi et al., 2003). The current results showed that CSE significantly increased the levels of IL‐6, IL‐1β, and hsCRP, as detected in the cell culture supernatant. The mRNA levels of the endothelial injury markers VCAM‐1 and ICAM‐1 in HPMECs were also significantly increased, which was consistent with the results of in vivo experiments. Furthermore, CSE activated the NF‐κB signaling pathway in HPMECs. These findings suggested that microvascular barrier dysfunction and pulmonary inflammation leading to systemic inflammation are critical links in COPD exacerbation of AS.

Tongxinluo is a traditional Chinese medicine composed of extractions or powders from various natural products. This treatment can strongly improve the function of vascular endothelial cells, reduce blood lipid content, inhibit plaque inflammation, and enhance the stability of vulnerable plaques. Studies have found that the BODE index (body mass index, airflow obstruction, dyspnea, and exercise capacity) and grade of patients with stable COPD treated with TXL capsules for 8 weeks were significantly lower than those before treatment and in conventional treatment groups, indicating that TXL capsules can improve the prognosis of patients with stable COPD (Jiang et al., 2017). Our previous studies demonstrated that TXL protects cardiac microvascular endothelial cells (CMECs) against reperfusion injury (Cui et al., 2014), significantly increases the microvascular density of ischemic brain tissue (Chang et al., 2012), and improves the microangiopathy of diabetic nephropathy (Wu et al., 2017), revealing that the common target of TXL in the treatment of major diseases of the heart and brain and diabetic nephropathy is microvascular endothelial cells. To the best of our knowledge, the present study, for the first time, clarified the protective effects of TXL on PMVECs in cigarette smoke combined with high‐fat diet‐induced COPD in mice with AS. Statins are a commonly used lipid‐lowering drug in clinical practice. Several studies have shown that statins also reduce inflammation (Ghobadi et al., 2014), inhibit oxidative stress (Ferreira et al., 2014), protect endothelial cells (Venturini et al., 2019), and stabilize plaques (Xu et al., 2018). The results showed that TXL combined with Ato inhibited serum lipid levels and aortic endothelial injury biomarkers in the COPD + AS group mice, reduced the expression of systemic inflammatory biomarkers, and inhibited the formation of atherosclerotic plaques better than Ato or TXL alone. Thus, TXL can enhance the antiatherosclerotic effect of Ato. On the anti‐inflammatory side, Ato reduces the production of inflammatory mediators by reducing lipid production (Vadali & Post, 2014). In this study, we observed that the pathomorphological and functional improvements were mainly due to a restoration of the decrease in the influx of inflammatory cells (macrophages and neutrophils) with a consequent reduction in the release of pulmonary inflammatory mediators (IL‐1β, TNF‐α) and inhibition of systemic inflammation. Another previous study showed that Ato can improve lung function in mice with emphysema induced by smoke exposure (Pinho‐Ribeiro et al., 2017). The Ato or TXL treatment groups showed improvement in pulmonary mechanical parameters. After TXL–Ato combined treatment, the improvement in lung inflammation and lung function in mice was more significant than that of the Ato group, suggesting that TXL enhances the improvement mediated by Ato in lung inflammation and lung function. Herein, we observed that TXL increases the expression of Tj proteins (VE‐cadherin and β‐catenin) and reduces pulmonary microvascular hyperpermeability, and Ato upregulates Tj protein expression but has no significant effect on pulmonary microvascular hyperpermeability in the COPD+AS group mice. Notably, the TXL–Ato combined treatment significantly reduced pulmonary microvascular hyperpermeability and increased the expression of Tj proteins in mice treated with smoking combined with a high‐fat diet. Furthermore, TXL enhanced the protective effect of Ato on the pulmonary microvascular barrier. Therefore, Ato may reduce lung inflammation rather than protect the pulmonary microvascular barrier and thus delay the decline in lung function. TXL may inhibit pulmonary inflammation and trigger systemic inflammation through the protective effect of the pulmonary microvascular barrier, thereby reducing the decline in lung function. This phenomenon was confirmed in vitro: the cells treated with TXL displayed significantly decreased pulmonary microvascular permeability and showed upregulated expression of Tj proteins. Consistent with the in vivo results, TXL also protected the pulmonary microvascular barrier function of HPMECs induced by CSE. Furthermore, pretreatment with TXL not only decreased the levels of inflammatory markers (IL‐6, IL‐1β, hsCRP, VCAM‐1, and ICAM‐1) but also reduced the protein levels of NF‐қB. The results showed that TXL reduces the inflammatory response of HPMECs by protecting the function of the pulmonary microvascular barrier. In conclusion, through the protective effect of the pulmonary microvascular barrier, TXL inhibits pulmonary inflammation and systemic inflammation, thus enhancing the effect of Ato on COPD combined with AS.

Although the current findings were primarily obtained from high‐quality studies, there are several limitations: (1) a cigarette smoking group should be established to explore the key mechanism underlying high‐fat diet‐mediated aggravation of cigarette smoking‐induced COPD by observing the degree of damage to the lung microvascular barrier; (2) the signaling pathway by which TXL protects the pulmonary microvascular barrier should be further explored to clarify the drug intervention target of TXL.

## 5 CONCLUSIONS

Chronic obstructive pulmonary disease (COPD) aggravates AS, and the mechanism is the destruction of pulmonary microvascular barrier function; thus, lung inflammation triggers systemic inflammation. TXL enhances the antiatherosclerotic effect of Ato through the protective effect on the pulmonary microvascular barrier in COPD combined with AS. This combined treatment regimen has prospects in clinical applications.

## FUNDING INFORMATION

This work was supported by the National Key Research and Development Program (No. 2017YFC1700500) and Hebei Province Natural Science Foundation (No. H2018106043; H2021106032).

## CONFLICT OF INTEREST

The authors declare that they do not have any conflict of interest.

## ETHICS STATEMENT

This study was approved by the ethical guidelines of the Ethics Committee of Hebei Yiling Pharmaceutical Research Institute (No. N2020051), and the experimental procedures were conducted in accordance with the ethical standards of the Care and Use of Laboratory Animals for biomedical research published by National Institutes of Health (No. 85‐23, revised 1996).
